# Children’s intervention participation is associated with more positive beliefs towards active school transportation among parents

**DOI:** 10.1093/heapro/daad016

**Published:** 2023-03-18

**Authors:** Hanna Forsberg, Stina Rutberg, Lars Nyberg, Anna-Karin Lindqvist

**Affiliations:** Department of Health, Education and Technology, Luleå University of Technology, SE 97187 Luleå, Sweden; Department of Health, Education and Technology, Luleå University of Technology, SE 97187 Luleå, Sweden; Department of Health, Education and Technology, Luleå University of Technology, SE 97187 Luleå, Sweden; Department of Health, Education and Technology, Luleå University of Technology, SE 97187 Luleå, Sweden

**Keywords:** active school transportation, children, intervention, parents, physical activity, theory of planned behaviour

## Abstract

Insufficient physical activity among children is a critical issue and health promoting initiatives are required to reverse this trend. In response to the current situation, a school-based intervention aiming to increase physical activity with the aid of active school transportation (AST) was implemented in one municipality in northern Sweden. By adopting the framework of the Theory of Planned Behavior, we aimed to analyse beliefs among parents whose children were or were not involved in the AST intervention. All municipality schools were included. There were 1024 responses from parents, comprising 610 who responded either ‘yes’ or ‘no’ to participating in the intervention. An adjusted linear regression analysis showed that children’s intervention participation was significantly associated with more positive beliefs towards AST among parents. These results indicates that it is possible to influence beliefs that are important in the parental decision-making process by the use of an AST intervention. Therefore, to make children’s active transport to school the more favorable choice for parents, it seems to be worthwhile to not only give children the opportunity to participate but also to involve parents and address their beliefs when designing interventions.

## INTRODUCTION

Insufficient physical activity among children is a critical issue worldwide (Guthold *et al*., 2020). Consequently, initiatives that promote physical activity are required (Ding *et al*., 2016). An overview of reviews recently concluded that active transport is an important way to attain the recommendations of daily physical activity (Prince *et al*., 2021). Thus, active school transportation (AST), that is, cycling or walking to school, has been suggested as a promising strategy to promote children’s physical activity (Larouche *et al*., 2014; Prince *et al*., 2021). Fostering such behaviours in the younger years is also important (Yang *et al*., 2013; Telama *et al*., 2014) and thus, early initiatives to reverse the declining trends in physical activity are required (Ding *et al*., 2016). From a health perspective, promoting cycling and walking is a good idea, because there is no minimum level required before it becomes beneficial (World Health Organisation, 2022). Mizdrak *et al.* (2019) concluded that interventions aiming at encouraging a shift from short car trips to cycling and walking is likely to be a cost-effective way to improve population health while reducing greenhouse gas emissions. Because parents are the ultimate decisions-makers of their children’s transportation choice to school, it is necessary to involve them in promoting AST and to increase their awareness regarding the importance of such travel behaviour (Aranda-Balboa *et al*., 2019). The World Health Organisation (WHO 2022) suggested that promoting cycling and walking in its broadest sense concerns efforts that are aimed at shifting the perceptions of key factors related to travel behaviour such as, availability, social norms, convenience, attractiveness, and safety. They argue that a shift in these key factors may often be seen as modest. However, over time, the successful and sustainable promotion of such behaviours should be seen as the sum of many, often small improvements, each of them contributing to the formation of making active travel a bit more favourable ‘*to some people, for some trips, in some situations*’ *p. 17* (World Health Organisation, 2022).

In response to insufficient physical activity levels, a school-based health promoting intervention aiming to increase physical activity among children with the aid of AST was developed and implemented in a municipality in northern Sweden 2016. The intervention and its development which is described elsewhere (Lindqvist and Rutberg, 2018), were underpinned by social cognitive theory (SCT), empowerment, and gamification elements, targeting behaviour change in both children and parents, and is delivered by teachers in the school setting. The SCT was chosen as a theoretical framework because it is a frequently used theory in health promotion and has previously shown to be successful in promoting physical activity behaviour in interventions (McGoey *et al*., 2015). The concept of empowerment was used for the involvement of end-users throughout the whole process (Tengland, 2012). Gamification, that is the use of game-design in a non-game context, were included as a motivation component for children (Deterding *et al*., 2011). Several studies investigating children’s, teachers’, and parents’, experiences regarding the intervention have been conducted. One of these studies found that parents changed their attitudes in a positive way when they experienced that their children could use AST safely (Rutberg and Lindqvist, 2018). Another qualitative follow-up study demonstrated that children who had participated in the intervention developed a habit of using AST (Savolainen *et al.,* 2020).

To advance the understanding of the behavioural aspects concerning transportation decisions, (Forward, 2014, 2019) including children’s active travel to school (Murtagh *et al*., 2012) previous studies have used the Theory of Planned Behaviour (TPB) (Ajzen, 1991). The TPB suggests that behaviour is determined by peoples’ intentions, which in turn is a function of their attitudes, subjective norms, and perceived behavioural control (Ajzen, 1991). These three constructs are formed by three indirect measures which are, peoples’ behavioural beliefs, normative beliefs, and control beliefs. The indirect measures provide a more comprehensive understanding of the factors involved in motivating a certain behaviour (Ajzen, 1991; Forward, 2014, 2019). In recent years, an additional construct has been added to the TPB, and this is the descriptive norm, because it is thought that the subjective norm might be too narrow of a concept for norms (Rivis and Sheeran, 2003). In contrast to the subjective norm, which refers to what others think of me performing the behaviour, the descriptive norm captures the perception of what others do, thereby offering additional information to the subjective norm. Finally, a recent scoping review concluded that there are few studies investigating how AST interventions influence key factors related to the parental decision-making process (Savolainen *et al.*, submitted for publication). Therefore, by adopting the framework of the TPB, we aimed to analyse beliefs among parents whose children either were or were not involved in the AST intervention.

## METHODS

### Context

This study was conducted in northern Sweden, in a municipality with approximately 80 000 inhabitants. All municipal schools were included, which were located in both urban and rural areas. The climate in northern Sweden is characterized by a long cold snowy winter and a short spring, summer, and autumn.

### Intervention

This 4-week intervention started initially with the head of school giving principals at each municipal school information about the intervention. In turn, principals assigned the intervention to teachers. Information included encouragement, but not obligation, to launch the intervention each year, in grade 2 and 5. However, teachers has been free to implement the intervention in whichever grade they found most suitable. Based on the method (Lindqvist and Rutberg, 2018) the intervention consisted of a refined framework involving five steps. The first step involved parents by giving information and encouraging discussion about the intervention in a parental meeting. This included the intervention constituent activities, the benefits of AST and the parents giving their informed consent for children to participate. In the second step, children were assigned to find out more about the benefits of using AST. In the third step, teachers decided assignments related to the curriculum, for example, finding geometrical shapes on the way to school as a mathematic assignment. In the fourth step, the four-week period of encouraging AST, for part of or their entire trip to school started. Through the use of gamification, children measured AST and solved the weekly assignments that were previously chosen by the teachers. In the fifth step, accomplishments were celebrated.

### Procedure and measures

We developed a questionnaire based on the TPB to understand parents´ intentions to let their child use AST (PILCAST). The development and initial validation of the questionnaire showed acceptable validity and reliability (Forsberg *et al.,* 2021). The questionnaire covered the theoretical constructs of the TPB and treated cycling and walking to school as separate behaviours. The following scenario was presented to parents, ensuring that they responded to the same situation: ‘*Imagine your child cycling or walking to school this time a year when the weather is nice and clear. Please, try to consider the following statements even if your child does not cycle or walk to school*’.

#### Intention

The following two questions were used to measure intention ‘*In the next 3 upcoming weeks, I intend to let my child cycle/walk to school*’ and ‘*In the next 3 upcoming weeks, I plan to let my child cycle/ walk to school*’. Answer options ranged from 1 = Strongly disagree to 7 = Strongly agree.

#### Attitudes

A combination of behavioural beliefs and outcome evaluation were used to measure positive and negative attitudes, thus capturing the consequences of the behaviour and the evaluation of such consequences (Ajzen, 1991). Positive attitudes consisted of three behavioural beliefs and it´s corresponding outcome evaluation: increased independency, improved concentration in school, and improved health. Negative attitudes were measured using two behavioural beliefs and its outcome evaluation items: to cumbersome preparations and the trip takes too long. Each question for behavioural beliefs started with the following statement: ‘*If you would let your child cycle/walk to school, how much would you agree to the following statement?*’. Answer options ranged from 1 = Strongly disagree to 7 = Strongly agree. Outcome evaluation used the same items but were rephrased based on how important they were (1 = Not very important to 7= Very important).

#### Social norms

Subjective norms were measured using three items and each question started with the following: ‘*What do you think others in your immediate vicinity would think about you, letting your child cycle/walk to school?*’. The three items referred to friends, parents, and co-workers/fellow students. Answer options ranged from 1 = Completely unacceptable to 7 = Completely acceptable and I don’t know/Does not apply to me. Each question for the descriptive norm started with the following: ‘*If you consider those in your immediate vicinity who have children under the age of 12, do they let their children cycle/walk to school?*’. The three questions referred to the same kind of groups as the subjective norm and answer options ranged from 1 = Strongly disagree to 7 = Strongly agree and I don’t know/Does not apply to me.

#### Impeding and facilitating perceived behavioural control

To measure impeding and facilitating perceived behavioural control, a combination of control belief strength and power was used, representing the likelihood of the factor being present and the perceived power of these factors (Ajzen, 1991). The control belief strength questions started with ‘*How much would the following impede/facilitate you to let your child cycle/walk to school?*’. Three items measured impeding control belief strength: crossing an unattended pedestrian crossing, crossing a major road, and travel along roads with higher speeds than 40 km/h. Four items measured facilitating control belief strength: trusting the child, child being able to navigate, safe environment, and separate walking/cycling lanes. Answer options ranged from 1 = Very little to 7 = Very much. Impeding and facilitating control belief power were assessed with the same items but were rephrased based on how they applied to their situation. Answer options ranged from 1 = Strongly disagree to 7 = Strongly agree. The combinational constructs were used as described previously by Ajzen (Ajzen, 1991).

Data collection was conducted during September, by administering a web-based questionnaire to parents with children in school years 1–6 in the present municipality. Parents were able to respond to the questionnaire for a total of 25 days, and after 14 days a reminder to respond was sent out.

### Participants

There were 1024 responses received from parents, comprising 610 responding either ‘yes’ or ‘no’ to the question: *‘Are you aware if your child has participated in the AST intervention- I move forward?’*. The 414 who responded ‘I don’t know’ were not included in the analysis. Among the 610 included responses, the ‘yes’ group was somewhat larger, (*n* = 324, 56.1%) than the ‘no’ group, (*n* = 268, 43.9%) (Table 1). A large proportion of the sample consisted of women, and the parents almost exclusively originated from Sweden and the Nordic countries. Additionally, most parents in the sample reported having a higher education. The most common age was 40–49 years, in both groups. Although there were slightly more boys in the ‘yes’ group than in the ‘no’ group, the distribution of the children’s gender was equal overall for girls and boys. However, there were few children in the group reported as ‘other’. Most children had a distance of approximately 0–2 km to school, and they were evenly distributed across academic school years 1–6. A fairly large proportion of children in academic school year 1, were in the ‘no’ group (*n* = 104, 86.7%) compared to the ‘yes’ group (*n* = 16, 13.3%).

**Table 1:** Characteristics of the participants

	Yes 342 (56.1)	No 268 (43.9)	Total 610 (100)
	*n* (%)	*n* (%)	*n* (%)
Gender of parent			
Women	286 (83.6)	200 (74.6)	486 (79.7)
Men	56 (16.4)	68 (25.4)	124 (20.3)
Other	–	–	–
Age of parent			
18–29	3 (0.9)	12 (4.5)	15 (2.5)
30–39	123 (36.0)	94 (35.1)	217 (35.6)
40–49	195 (57.0)	139 (51.9)	334 (54.8)
50–55>	21 (6.1)	23 (8.6)	44 (7.2)
Ethnicity of parent			
Sweden and the Nordic countries	328 (95.9)	257 (95.9)	585 (95.9)
Non-Nordic countries	14 (4.1)	11 (4.1)	25 (4.1)
Education of parent			
Lower (elementary, secondary school or other)	66 (19.3)	94 (35.1)	160 (26.2)
Higher (higher education institution)	276 (80.7)	174 (64.9)	450 (73.8)
Gender of child			
Girl	153 (44.7)	140 (52.2)	293 (48.0)
Boy	187 (54.7)	128 (47.8)	315 (51.6)
Other	2 (0.6)	–	2 (0.3)
Academic school year of child			
Year 1	16 (4.7)	104 (38.8)	120 (19.7)
Year 2	59 (17.3)	39 (14.6)	98 (16.1)
Year 3	94 (27.5)	20 (7.5)	114 (18.7)
Year 4	50 (14.6)	46 (17.2)	96 (15.7)
Year 5	57 (16.7)	25 (9.3)	82 (13.4)
Year 6	66 (19.3)	34 (12.7)	100 (16.4)
Distance to school			
0–1 km	156 (45.6)	122 (45.5)	278 (45.6)
1.1–2.0 km	102 (29.8)	72 (26.9)	174 (28.5)
2.1–3.0 km	41 (12.0)	36 (13.4)	77 (12.6)
3.1–4.0 km	11 (3.2)	13 (4.9)	24 (3.9)
4.1–5.0 km	5 (1.5)	7 (2.6)	12 (2.0)
5.1–10.0 km	18 (5.3)	10 (3.7)	28 (4.6)
10.0> km	9 (2.6)	8 (3.0)	17 (2.8)

### Data analyses

To analyse beliefs among the parents of children who were or were not involved in the AST intervention, we performed separate analyses for each of the of the TPB constructs. These analyses was performed using bivariate (non-adjusted) *t*-tests, and adjusted for the academic school year and distance to school (Rothman *et al*., 2018; Saleme and Pang, 2021) using multiple regression analyses. When Levene’s test was significant, the test that did not assume equal variances, was used, *p* < 0.05 was considered to be significant. If *p* was close to the significance level, further analysis was conducted using the Welch’s test. The answer option ‘I don’t know/Does not apply to me’ was treated as missing data for the analyses. For the TPB cycling constructs 67 responded ‘I don’t know/Does not apply to me’ for the subjective norm, and 160 participants responded in this manner for the descriptive norm. The corresponding number of participants with this response for the TPB walking constructs was 75 and 175, respectively (data not shown). All analyses were conducted using IBM SPSS Statistics for windows, version 28.0 (IBM Corp., Armonk, NY, USA).

## RESULTS

Table 2 shows that across all the TPB cycling and walking constructs, the parents who participated in the intervention was moderately but significantly more positive towards AST than the non-intervention group. The TPB walking construct impeding perceived behavioural control were significant at the level of *p* = 0.048, and therefore a Welch test was performed, showing the same significance level (*p* = 0.048) (data not shown).

**Table 2:** TPB constructs across the ‘yes’ and ‘no’ groups for participation in the AST intervention

Variable	Cycle to school			Walk to school		
	Yes	No	*p*	Yes	No	*p*
Intention^†^	5.6 (2.1)	4.8 (2.6)	< 0.001	3.6 (2.7)	2.9 (2.5)	0.002
Positive attitude ^††,††,*^	19.8 (5.1)	18.1 (6.0)	< 0.001	19.8 (5.1)^*^	18.1 (6.0)^*^	< 0.001
Negative attitude^†††^	4.3 (4.2)	5.7 (6.0)	< 0.001	5.5 (5.1)	7.0 (6.7)	0.002
Subjective norm^†^	6.2 (1.4)	5.6 (1.8)	< 0.001	6.0 (1.6)	5.5 (2.0)	< 0.001
Descriptive norm^†^	5.4 (1.4)	4.8 (1.8)	< 0.001	5.3 (1.5)	4.6 (1.9)	< 0.001
Impeding perceived behavioural control^†††^	9.5 (6.6)	10.7 (7.6)	0.034	9.3 (6.7)	10.4 (7.4)	0.048
Facilitating perceived behavioural control^†††^	18.1 (5.4)	15.8 (6.3)	< 0.001	18.1 (5.4)	15.8 (6.3)	< 0.001

Independent *t*-test, showing means, standard deviation, and *p*-value.

Yes, intervention group; no, non-intervention group.

^†^Scale 1–7, ^††^scale 1.0–24.5, ^††,*^1.17–24.5, ^†††^scale 0.5–24.50 (a higher value always indicates a stronger belief).

In the unadjusted linear regression analyses (Tables 3 and 4) participation in the intervention was significantly associated with all the TPB cycling and walking constructs. Participation in the intervention was negatively associated with a negative attitude and impeding perceived behavioural control. In the second step when adjusting for the academic school year and distance to school (Table 3), participation in the intervention remained significantly associated with all the TPB cycling constructs except for impeding perceived behavioural control. Participation in the intervention was most strongly associated with a positive attitude, followed by the subjective norm, facilitating perceived behavioural control and descriptive norm.

**Table 3:** Linear regression with the TPB cycling constructs set as dependent

Dependent variable	Independent variables	Unadjusted			Adjusted			VIF	Model characteristics		
		*B*	*β*	*p*	*B*	*β*	*p*		Durbin Watson	Adjusted R^2^	*p*
Intention									1.889	0.296	< 0.001
	Intervention	0.752	0.160	< 0.001	0.459	0.098	0.006	1.087			
	Academic school year of child				0.270	0.200	< 0.001	1.100			
	Distance to school				0.777	0.509	< 0.001	1.013			
Positive attitude									1.963	0.048	< 0.001
	Intervention	1.721	0.154	< 0.001	1.750	0.156	< 0.001	1.087			
	Academic school year of child				−0.054	−0.017	0.683	1.100			
	Distance to school				0.607	0.167	< 0.001	1.013			
Negative attitude									2.024	0.327	< 0.001
	Intervention	−1.451	−0.141	< 0.001	−0.984	−0.096	0.006	1.087			
	Academic school year of child				−0.404	−0.136	< 0.001	1.100			
	Distance to school				−1.862	−0.556	< 0.001	1.013			
Subjective norm									1.955	0.197	< 0.001
	Intervention	0.541	0.163	< 0.001	0.399	0.120	0.003	1.096			
	Academic school year of child				0.066	0.069	0.087	1.108			
	Distance to school				0.459	0.423	< 0.001	1.022			
Descriptive norm									1.952	0.115	< 0.001
	Intervention	0.621	0.189	< 0.001	0.371	0.113	0.017	1.120			
	Academic school year of child				0.190	0.204	< 0.001	1.136			
	Distance to school				0.263	0.251	< 0.001	1.026			
Impeding perceived behavioural control									1.927	0.245	< 0.001
	Intervention	−1.243	−0.087	0.032	−0.794	−0.056	0.130	1.087			
	Academic school year of child				−0.369	−0.090	0.015	1.100			
	Distance to school				−2.292	−0.494	< 0.001	1.013			
Facilitating perceived behavioural control									2.034	0.241	< 0.001
	Intervention	2.242	0.187	< 0.001	1.432	0.119	0.001	1.087			
	Academic school year of child				0.764	0.222	< 0.001	1.100			
	Distance to school				1.676	0.430	< 0.001	1.013			

**Table 4:** Linear regression with the TPB walking constructs set as dependent

Dependent variable	Independent variables	Unadjusted			Adjusted			VIF	Model characteristics		
		*B*	*β*	*p*	*B*	*β*	*p*		Durbin Watson	Adjusted *R*^2^	*p*
Intention									1.898	0.175	< 0.001
	Intervention	0.639	0.122	0.002	0.370	0.071	0.066	1.087			
	Academic school year of child				0.250	0.166	< 0.001	1.100			
	Distance to school				0.665	0.391	< 0.001	1.013			
Positive attitude									1.971	0.054	< 0.001
	Intervention	1.737	0.155	< 0.001	1.756	0.157	<0.001	1.087			
	Academic school year of child				−0.046	−0.014	0.728	1.100			
	Distance to school				0.666	0.183	< 0.001	1.013			
Negative attitude									2.011	0.375	< 0.001
	Intervention	−1.508	−0.127	0.002	−1.058	−0.089	0.008	1.087			
	Academic school year of child				−0.368	−0.108	0.001	1.100			
	Distance to school				−2.342	−0.605	< 0.001	1.013			
Subjective norm									1.917	0.306	< 0.001
	Intervention	0.546	0.149	<0.001	0.374	0.102	0.007	1.092			
	Academic school year of child				0.071	0.068	0.074	1.101			
	Distance to school				0.647	0.541	< 0.001	1.018			
Descriptive norm									1.951	0.129	< 0.001
	Intervention	0.659	0.194	< 0.001	0.430	0.127	0.007	1.117			
	Academic school year of child				0.171	0.178	< 0.001	1.135			
	Distance to school				0.313	0.289	< 0.001	1.026			
Impeding perceived behavioural control									1.937	0.242	< 0.001
	Intervention	−1.150	−0.081	0.045	−0.726	−0.051	0.164	1.087			
	Academic school year of child				−0.344	−0.085	0.023	1.100			
	Distance to school				−2.261	−0.491	< 0.001	1.013			
Facilitating perceived behavioural control									2.027	0.240	< 0.001
	Intervention	2.260	0.189	< 0.001	1.472	0.123	< 0.001	1.087			
	Academic school year of child				0.740	0.215	< 0.001	1.100			
	Distance to school				1.680	0.431	< 0.001	1.013			

Regarding the TPB walking constructs, in the adjusted analysis participation in the intervention remained significantly associated with all the TPB constructs except intention and impeding perceived behavioural control in the adjusted analysis (Table 4). Participation in the intervention was mostly associated with a positive attitude. The next strongest associations were the descriptive norm, facilitating perceived behavioural control followed by the subjective norm. In summary, all regression models were statistically significant, and the explained variance ranged from 4.8 to 32.7% (TPB cycling models) and 5.4% to 37.5% (TPB walking models), respectively. Further, the analyses yielded low variance inflation factor (VIF) values and a Durbin Watson value approximately 2 in most cases, indicating that multicollinearity and intercorrelation were not a problem in the regression models (Tables 3 and 4).

## DISCUSSION

By adopting the framework of the TPB, we aimed to analyse beliefs among parents whose children were or were not involved in the AST intervention. The adjusted linear regression analysis showed that children’s intervention participation was significantly associated with more positive beliefs towards AST among parents. These results indicate that it is possible to influence beliefs that are important in the in the parental decision-making process by the use of an AST intervention.

The significance of influencing parents’ decisions concerning AST was recently highlighted in a review, because their beliefs are critical for how well school initiatives translates into children’s actual engagement in AST behaviours (Vasey *et al*., 2022). Similarly, a recent study concluded that interventions aiming to promote children’s independent mobility should focus on altering parents’ attitudes (Vlaar *et al*., 2019). Previous research also showed that parents’ perceptions of their child´s independent mobility are unlikely to change, if children are not given the opportunity to develop and practice skills for active travel (Crawford *et al*., 2017). In this study, a positive attitude was linked to a belief that the child would increase their independence, improve health and concentration in school. The association between participation in the intervention and a positive attitude might occur because the parents received information concerning the benefits of AST and because their child was given an opportunity to use AST. Similar results have been found elsewhere, suggesting that parents whose children had participated in an AST intervention became more aware of benefits such as health and wellbeing and other outcome expectations such as the child being on time (Huang *et al*., 2018; Rutberg and Lindqvist, 2018; Scharoun Benson *et al*., 2020). Although the historic discourse about active transport has mainly focussed on the disadvantages, such as risks and safety, more attention has recently been directed towards to the benefits (World Health Organisation, 2022). The importance of emphasising the benefits and positive outcomes of AST has been suggested as crucial when promoting these kinds of behaviours (World Health Organisation, 2022). This is also well supported since much evidence points in the direction of benefits outweighing the disadvantages of active travel (Mizdrak *et al*., 2019; World Health Organisation, 2022).

Further, the results of this study demonstrated that participation in the intervention was associated with subjective and descriptive norms concerning both cycling and walking to school. These results are consistent with a previous study demonstrating a shift in social norms among parents whose child had participated in a program aiming to encourage children to walk to school (Schuster *et al*., 2016a). According to Festinger’s theory of cognitive dissonance, people strive towards consistency between their beliefs and behaviours (Festinger, 1962). Consistent with this, Schuster and colleagues (2016a) suggested that parents whose children participate in AST interventions alter their social norms to be more consistent with their children using AST. The importance of social norms concerning the parental decision-making process related to AST has previously been reported by others (Schuster *et al*., 2016b; Saleme and Pang, 2021) and by us (Forsberg *et al.,* 2020, 2021). For example, we reported that parents’ were afraid to be considered irresponsible if they let their children independently use AST (Forsberg *et al.,* 2020). Similarly, Pynn *et al*., (2019) outlined in their study that parents were afraid of being judged as bad parents if they let their children get out on their own. However, parental social norms also withhold the potential to positively influence decisions. We reported previously that when parents perceive that others let their children use AST, they found inspiration to let their children use AST (Forsberg *et al.,* 2020). This means that when parents perceive that others let their children use AST, they become more inclined to let their children use AST. In line with these findings, Saleme and Pang (2021) also stressed the important role of social norms in overcoming perceived risks among parents. This is also in agreement with Ajzen (1991) who argued that different kind of beliefs will take on different importance across various types of behaviours. A reason for social norms being important for the execution of certain behaviours has previously been discussed. For example, Rivis and Sheeran (2003) argued that descriptive norm seems to take on importance when there is a perceived risk involved in the performance of the behaviour. However, for AST, much evidence suggest the same regarding the subjective norm, which is what others think about me as a parent letting my child use AST (Schuster *et al*., 2016a, 2016b; Forsberg *et al.*, 2020, 2021; Saleme and Pang, 2021). Thus, considering the prevailing ideals of parenting and making it more socially accepted among parents should be considered a crucial key factor in promoting AST. Additionally, as demonstrated in this research and by others (Schuster *et al*., 2016a), interventions seem to be suitable for such endeavours.

In the present study, impeding perceived behavioural control concerned only beliefs about the built environment such as crossing an unattended pedestrian crossing, crossing a major road and travel along roads with higher speeds than 40 km/h. Participation in the intervention in the final analysis was not associated with these beliefs, which concerned both cycling and walking to school. However, such beliefs might be hard to influence using a health promoting school-based AST intervention. Participation in the intervention remained significantly associated with facilitating perceived behavioural control, which referred to beliefs such as trusting the child, child being able to navigate, safe environment, and separate walking/cycling lanes. Previous research has concluded that interventions aiming at promoting independent mobility should focus on ‘modifiable factors’ such as children’s skills and competence and on easing parents traffic concerns while helping them to gain confidence in their children’s abilities (Riazi *et al*., 2019, 2022) Thus, promoting AST using interventions might be beneficial to increase the attention towards the facilitating factors.

Faulkner *et al.* (2010) highlighted the importance of addressing the issue of behavioural costs such as time and convenience because these were shown to be crucial in the parental decision-making process. In this study, participation in the intervention was negatively associated to a belief that the preparations for a trip to school would be too cumbersome and that the trip would take too long time. This applied to both cycling and walking to school and this association remained significant even when adjusting for the distance to school, which may indicate that such beliefs about AST might be stereotyped and not determined only by distance. The possibility that experience could influence stereotypical beliefs regarding different travel modes, was also reported in previous studies (Forward, 2019). Highlighting the importance of personal experience when adopting new travel behaviours.

Participation in the intervention was not associated with the intention to walk to school and impeding perceived behavioural control, nevertheless, it was associated with the other TPB walking constructs. Ajzen (1991) argues as a general rule that the more favourable beliefs people hold, the stronger the intention will be to perform the behaviour, and that people will act on these behavioural intentions when they get the opportunity to do so. This means that favourable beliefs will be a prerequisite for people to act and that they will probably do so whenever they get an opportunity. For example, as this study was conducted during bare ground season, it is reasonable to assume that parents may act on their behavioural beliefs regarding walking to school when the conditions for active transport change during the wintertime.

The findings from this study needs to be interpreted in the light of some limitations. The cross-sectional nature of this study means that the results are associations only and that conclusions about causality cannot be drawn. We also know nothing about parents’ beliefs before participating in the intervention. However, the results of this study provide direction and support that it is worthwhile to conduct replicate studies including other more sophisticated study designs to overcome the limitations mentioned in the data. It is also likely that many of the participants who responded ‘I don’t know’ to the question about participation did not participate in the intervention, but we do not know that for sure. Thus, it was reasonable to exclude them from the analysis, but the results need to be interpreted with this in mind. In this study, most participants were women and previous studies within the AST area have shown similar patterns (Mandic *et al*., 2020). To compensate for such shortcomings in the data and to better capture parental beliefs, it has been suggested that future studies should address this issue. Finally, limitations notwithstanding, this study showed some encouraging results, demonstrating that children’s intervention participation is associated with more positive beliefs towards AST among parents.

## CONCLUSION

The results of this study showed that children’s intervention participation was significantly associated with more positive beliefs towards AST among parents. This indicates that it is possible to influence beliefs that are important in the parental decision-making process by the use of an AST intervention. Therefore, to make children’s active transport to school the more favourable choice for parents, it seems to be worthwhile to not only give children the opportunity to participate but also to involve parents and address their beliefs when designing interventions.
